# Association of serum beta hCG levels in women with palpable malignant breast lesions

**DOI:** 10.1038/s41598-023-40056-1

**Published:** 2023-08-14

**Authors:** Ashfaque Mohammed, Talha Ahmed, Rahul R. Bhat, Esha Mallik, Aashika Arulprakasam

**Affiliations:** Department of General Surgery, Kasturba Medical College, Mangalore, Manipal Academy of Higher Education, Manipal, India

**Keywords:** Cancer, Molecular biology, Biomarkers, Oncology

## Abstract

This study aims to determine whether serum Beta hCG can be used as a tumour marker in Breast malignancies. The objective of this study is to evaluate the serum Beta hCG in various stages of breast carcinoma and to correlate its level with disease severity and prognosis. Cross sectional analytical study of assessing serum Beta hCG in 200 patients with palpable breast malignancies at hospitals in urban Mangalore, India. In our study there was *No* increase in serum Beta hCG, in women with breast malignancies, but there was a pattern amongst the negative results. A Beta hCG of < 5mIU/mL is taken as negative, but in our study of 200 individuals, a mean value of 2mIU/mL was used as differentiation between low and high risk individuals. With our study we tried to correlate the value of Beta hCG with malignant breast lesions, and even though women with such lesions did not have a value of > 5mIU/mL, we found substantial evidence that women who had a value of > 2mIU/mL had a more advanced disease, be it in terms of staging, and comparing it with markers like ki67. A direct correlation between Beta hCG and severity of the disease in terms of staging was proved, hereby directly affecting the outcome of patients. Higher the level of Beta hCG, graver the prognosis. Even though Beta hCG cannot be used as tumour marker, it can be used to prognosticate the severity in women with palpable breast malignancies.

## Introduction

Human chorionic gonadotrophin (hCG) belongs to the glycoprotein group of hormones and is made of two subunits—the alpha (glycoprotein family) and beta (receptor specific) subunits. These are two non-covalently bound and glycosylated subunits^1^.

Beta human chorionic gonadotrophin is most commonly used for the detection of pregnancy. However, research has shown that its levels are also known to be elevated in entities such as tumours of the testes and ovary (germ cell tumours) and in gestational trophoblastic disease. Beta hCG is also known to be detected in tumours of pancreas, liver, stomach, cervix, and breast. Some studies have also found that Beta hCG levels seem to be elevated in breast related lesions^1–5^.

Numerous molecular markers already discovered and are known to affect breast cancer outcomes include Estrogen Receptor (ER), Progesterone Receptor (PR) from the steroid hormone receptor pathway and human epidermal growth factor in the HER family.

This study focuses on the evaluation of Beta hCG in malignant breast lesions and correlating the levels with various tumour stages, for a better understanding of its relationship with breast tissue and to analyze the possibility of using it in the diagnosis and management of breast malignancies. All patients with palpable breast malignancies were investigated further by evaluating their serum Beta hCG levels to evaluate if there is a correlational increase in these levels in these patients.

## Materials and methods

### Population and study design

This cross-sectional study of the analytical study type was conducted in urban Mangalore Hospitals affiliated to Kasturba Medical College, Mangalore.

It included patients of all tumour stages as assessed on clinical examination. Patients were subcategorized according to their TNM stage and further analysis was done regarding the Beta hCG presence according to tumour stage. The study was conducted over the period of 2 years from November 2018 to June 2020. Consent was taken from patients prior to their participation in the study along with informing their bystanders about the study. They were also given the assurance that the study would, in no way, have any impact on their course of treatment. All experimental protocols were approved by our ethical organization namely the “Kasturba Medical College, Mangalore – Institutional Ethics Committee, Manipal Academy of Higher Education (Reg. no. ECR/541/Inst/KA/2014/RR-20)” bearing number IEC KMC MLR 10-18/368.

### Inclusion criteria

All patients in the age group of 18–65 years with malignant breast lesions presenting with a palpable breast lump on clinical examination, regardless of their axillary lymph node status were included in the study.

### Exclusion criteria

Patients who were pregnant at the time of presentations, had a concurrent malignancy of any other organ such as ovarian cancer, choriocarcinoma and other Beta hCG secreting lesions, had a benign breast lesion or belonged to the age group of < 18 and > 65 years were excluded from the study.

### Description of procedure

Blood was drawn from each patient to measure serum beta hCG levels and several comparisons were thus generated in terms of:

- Differences in Beta hCG levels according to various stages of breast cancer in terms of TNM staging.
- Differences in Beta hCG levels according to age of the patient with malignant lesion
- Differences in Beta hCG levels between the different histological types of breast cancer.

All the above-mentioned methods were carried out in accordance with the relevant guidelines and regulations as dictated by the ethical and scientific committee of “Kasturba Medical College, Mangalore – Institutional Ethics Committee, Manipal Academy of Higher Education (Reg. no. ECR/541/Inst/KA/2014/RR-20)”.

### Informed consent

Written informed consent was obtained from all the patients for publication of this case series and accompanying images. A copy of the written consent is available for review by the Editor-in-Chief of this journal on request.

## Results

With our study sample of 200 women with malignant palpable breast lesions the various resultant outcomes and their correlation with various parameters of Age, History, Staging, Tumour markers and Histopathalogical types have been depicted in the form of charts and pie graphs. A statistical analysis was done to correlate the value of Beta hCG. We split the value of Beta hCG to values of < 0.7mIU/mL, 0.7–2.0mIU/mL and > 2mIU/mL to obtain a pattern amongst the results.

Table 1 gives a summary of our collected data. (Table 1) We found that amongst the 200 women subjected to serum Beta hCG test, all of them had a value of < 5mIU/mL, thereby giving a Negative result.

**Table 1:** Overview of all data.

		Count
Age	40 and below	12
	41–50	63
	51–60	77
	Above 60	48
History	Lump in B/L breast	1
	Lump in left beast	99
	Lump in right breast	100
Family history	Present	21
	Absent	179
Duration	< 12 months	124
	12–24 months	76
Histological diagnosis	Ductal carcinoma insitu	21
	Infiltrating ductal carcinoma	97
	Inflammatory carcinoma breast	3
	Invasive lobular carcinoma	44
	Invasive medullary carcinoma	5
	Invasive papillary carcinoma	5
	Invasive tubular carcinoma	5
	Lobular carcinoma in situ	20
ER	Positive	113
	Negative	87
PR	Positive	111
	Negative	89
Her2neu	Positive	90
	Negative	110
Ki67	High risk (≥ 15%)	116
	Low risk (< 15%)	84
T	1	20
	2	61
	3	79
	4	40
N	0	95
	1	61
	2	43
	3	1
Stage	I	19
	II	86
	III	69
	IV	26
Beta hCG Value (mIU/mL)	> 1.1	145
	< = 1.1	55
Beta hCG Value (mIU/mL)	< 0.7	49
	0.7–2.0	104
	> 2.0	47
Result	Negative	200

To establish a pattern amongst the negative result, we tried to split the value of Beta hCG into three groups based on the mean value. Hence patients were split into three groups based on the value of beta hCG as < 0.7mIU/mL, 0.7–2.0mIU/mL and > 2mIU/mL. Patients were then categorized into the same and results were tabulated.

### Co-relation with stage

**Figure 1:**
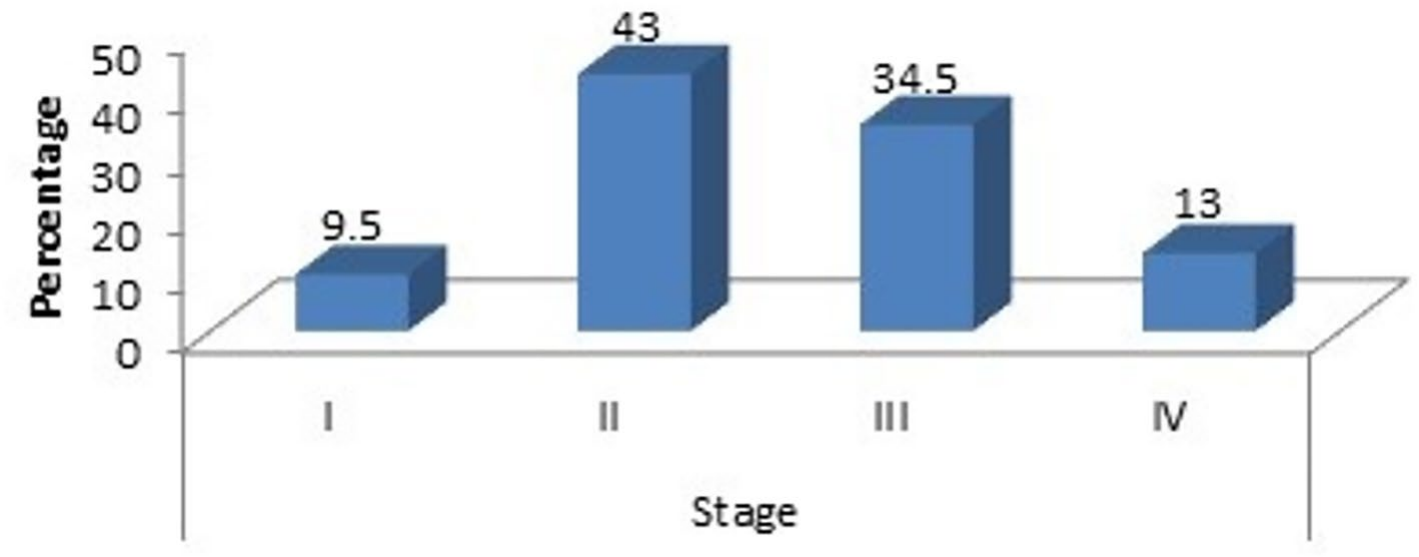
Correlation with stage of breast carcinoma.

Serum beta hCG levels were found to have a highly significant association with the stage of breast carcinoma, with beta hCG levels showing a rising trend with progressive worsening of stage of disease. Of the 19 women with stage 1 carcinoma, none showed a beta hCG value of > 2mIU/mL whereas, out of the 26 women with stage 4 disease, 76.9% had a beta hCG value of > 2mIU/mL, and the p value was found to be 0.00 which is highly significant (Fig. 1).

### Co-relation with histological diagnosis

Amongst the 200 women evaluated, 97 (48.5%) had infiltrating ductal carcinoma, 44 (22%) invasive lobular carcinoma, 21 (10.5%) had ductal carcinoma in situ, 20 (10%) had lobular carcinoma in situ, 5 (2.5%) invasive medullary carcinoma, 5 (2.5%) invasive papillary carcinoma, 5 (2.5%) invasive tubular carcinoma and 3 (1.5%) were cases of inflammatory carcinoma breast, with a p value of 0.078. Hence, there is no correlation between serum beta hCG level and histopathological subtype of carcinoma.

### Correlation with Ki-67

**Figure 2:**
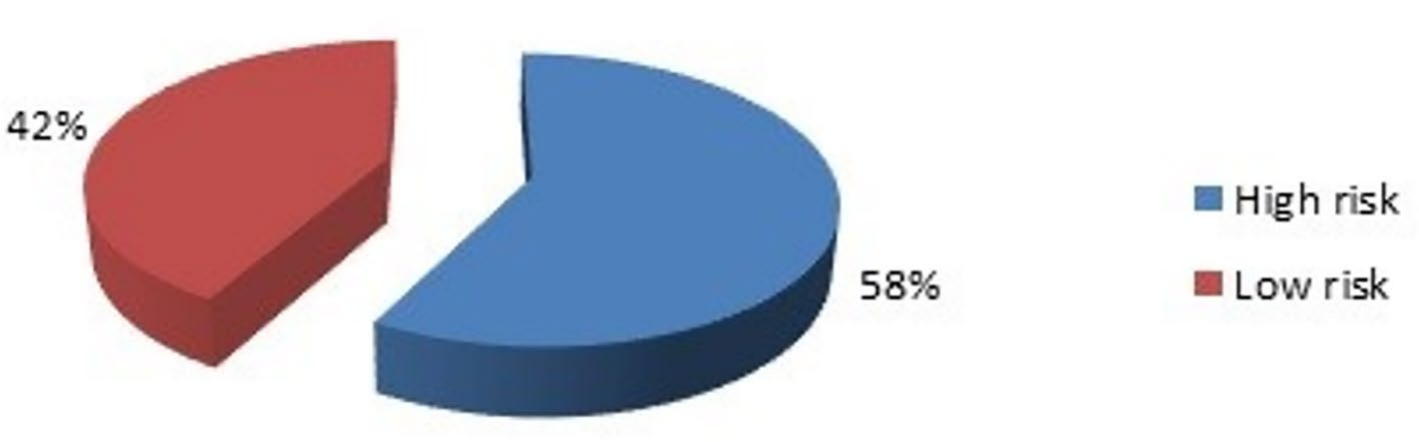
Correlation with ki67 status of patients.

A significant association was also seen between serum beta hCG levels and tumour marker ki67, with 30.2% of women with ki67 value ≥ 15% (high risk) showing a serum beta hCG of > 2mIU/mL with a *p* value of 0.033 (Fig. 2).

### Correlation with T and N stage

**Figure 3:**
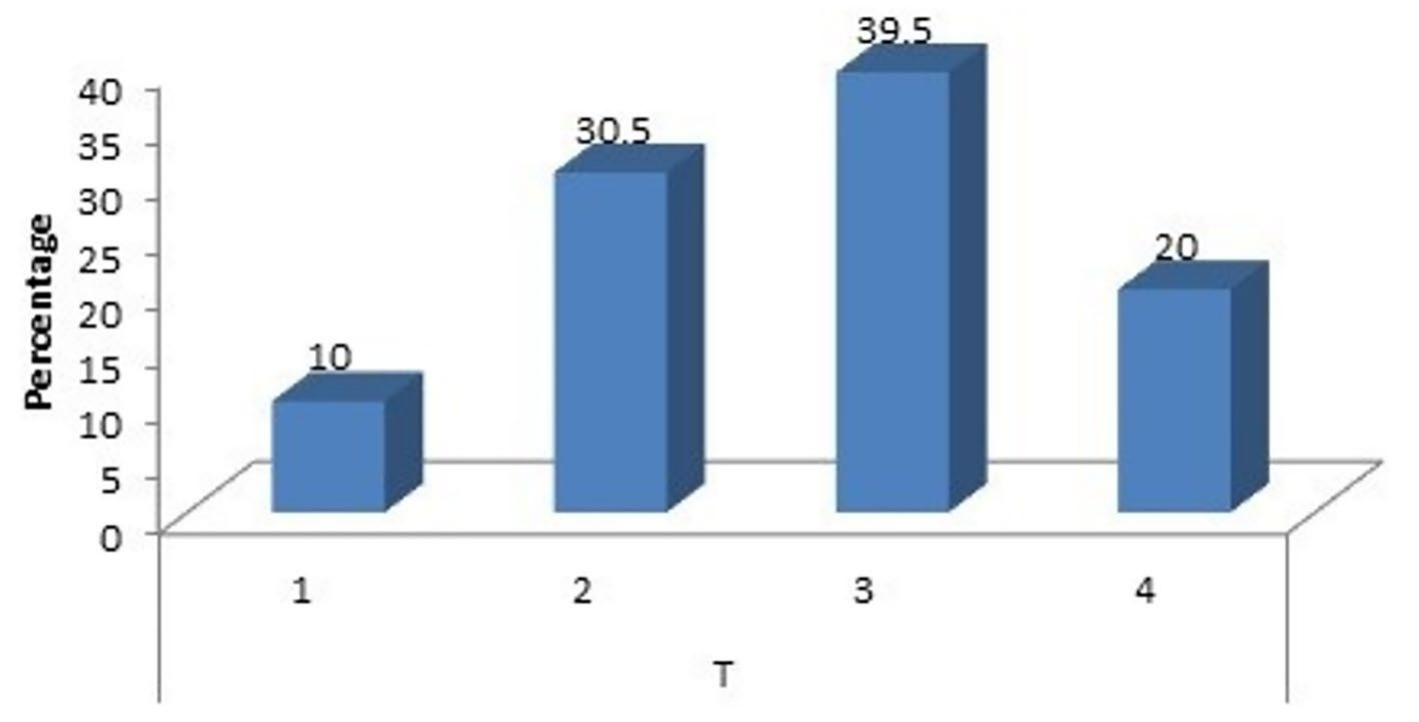
Correlation with T stage.

When we consider the T stage of the patient (Fig. 3), we found that 20 females were T1 stage, 61 were T2, 79 were T3 and 40 were T4 stage and when statistically analyzing the data 13(21.3%), 22(27.8%) and 12(30%) of the T2, T3 and T4 females had a beta hCG value of > 2mIU/mL, with a p value of 0.006, thereby leading to the inference that value of serum beta hCG in T stage of the disease is highly significant.

**Figure 4:**
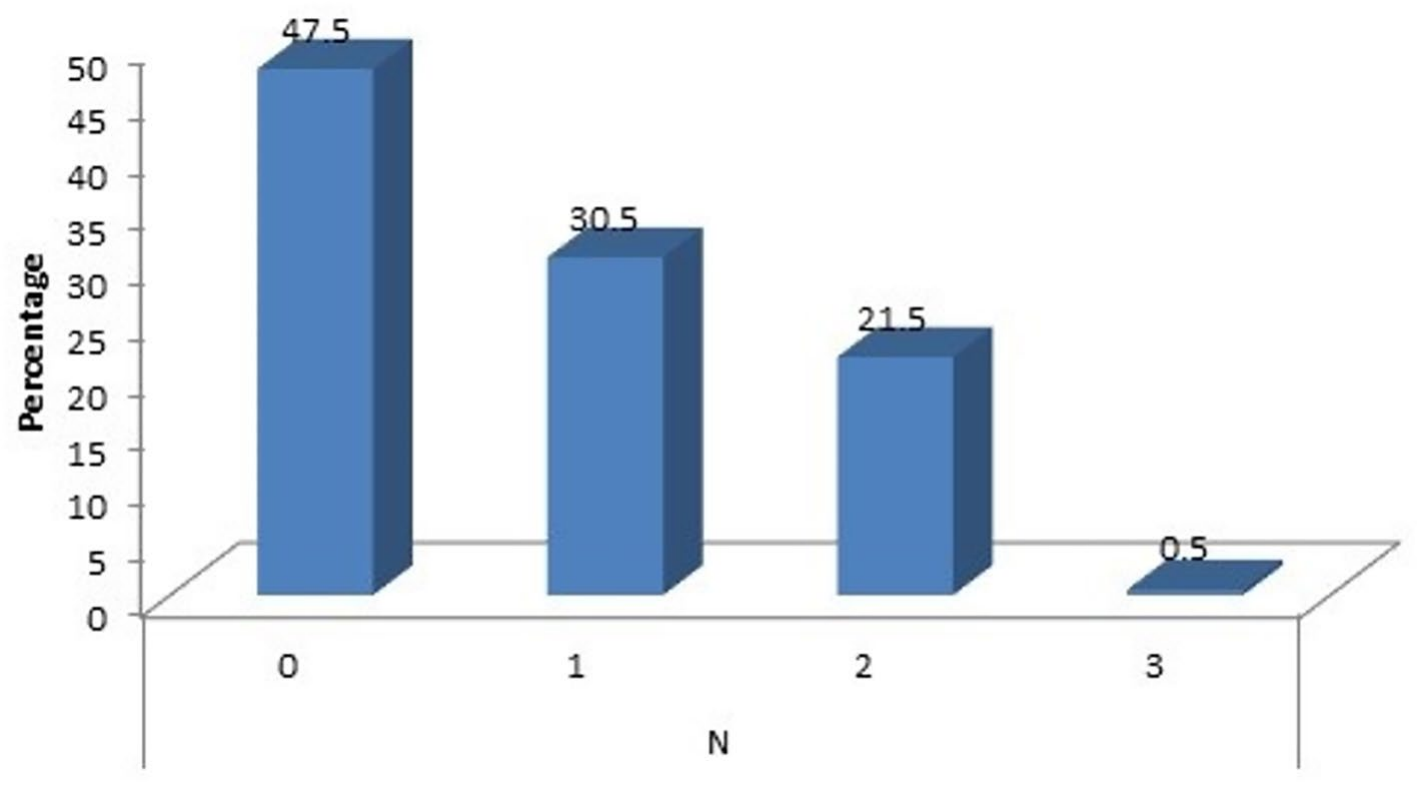
Correlation with N stage.

When analyzing the N stage of the disease (Fig. 4), 95 women had N0 disease, 61 of them had N1 disease, 43 had N2 disease and 1 had N3 disease. Of them 16(16.8%), 21(34.4%), 9(20.9%) and 1(100%) respectively had a beta hCG value of > 2mIU/mL with a *p* value of 0.011 which is significant.

### Co-relation with M stage

**Figure 5:**
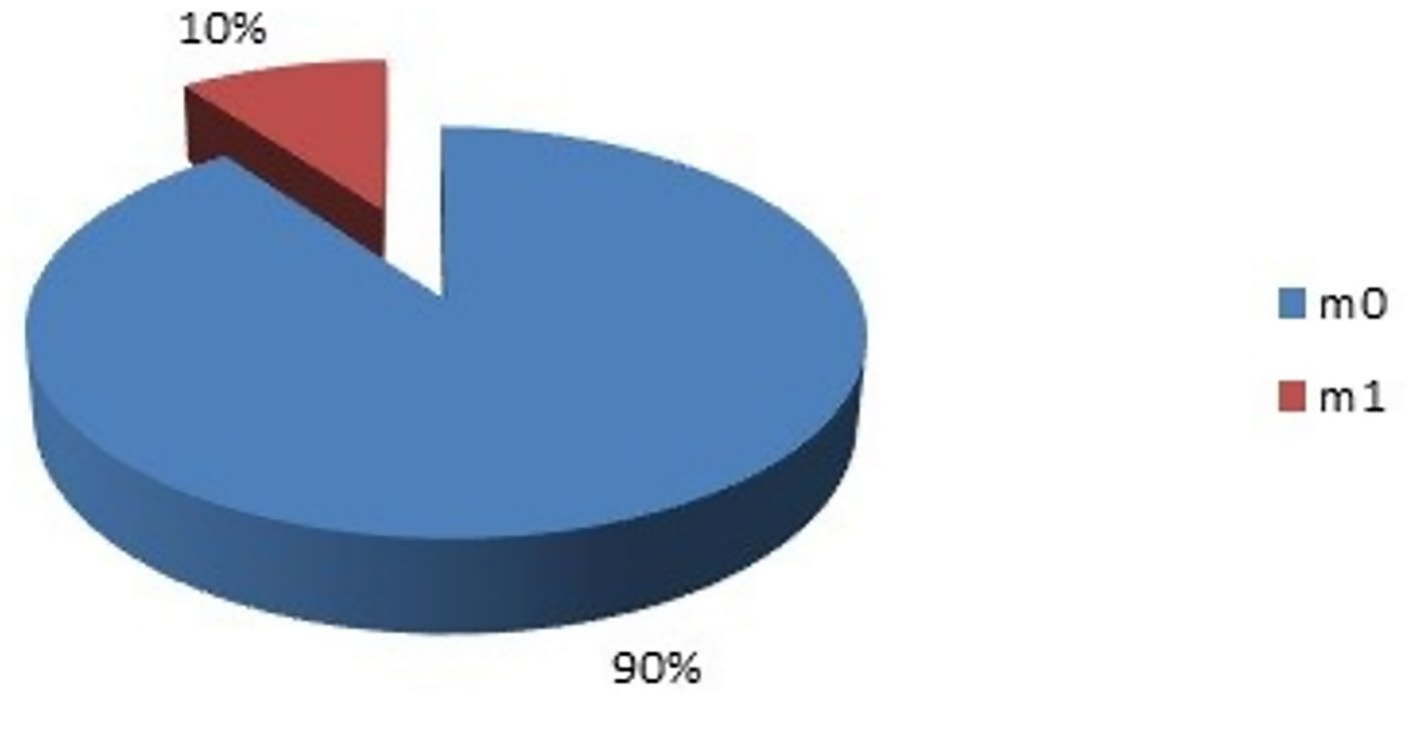
Correlation in patients with metastatic disease.

20 women had metastatic disease and all of them had a serum beta hCG level of > 2mIU/mL with a *p* value of 0.004 which was highly significant. With this result we can infer that metastatic disease has a direct impact on the beta hCG level, irrespective of the site of metastasis (Fig. 5).

## Discussion

Breast cancer is the most commonly occurring cancer in women and accounts for 14% of all cancers in Indian women. In India, the disease begins to rise in early 30 s and peaks in the fifth to sixth decade of life^6^. In India, there is an alarming increase in trend towards premenstrual age group being affected, with almost 48% patients being below 50 years of age. Most people in developing countries such as India present only when symptomatic, when stage 2B and beyond, so their survival rates are less than their western counterparts. On an average, out of 100 women with breast cancer in the US, 89 are likely to survive for 5 years, but in India it is estimated that this figure is barely 60%^6^. This high burden of disease makes it pertinent to recognize the disease at an early stage and initiate appropriate treatment.

ER, PR and Her2neu, Ki-67 are standard prognostic and tumour markers used in breast cancer. Based on the above-mentioned molecular markers, breast carcinoma can be divided into 4 molecular subtypes, namely,

- *Luminal A type*: These type tumors are low grade, carry the best prognosis and grow slowly. ER positivity and PR positivity are seen in 80% and 65% of breast cancers, respectively. These types of tumors grow well in the presence of such hormones. Hence, targeted hormonal therapy in such tumors show excellent response.
- *Luminal B type*: These types of tumors are faster growing than Luminal A tumors and carry a worse prognosis when compared to Luminal A. Drugs targeted against Her2neu can be used for the treatment of these tumours.
- *Her2neu positive type*: They comprise 20% of all breast tumors. They are fast growing, present early and are aggressive with a high turnover rate. Drugs targeted against Her2neu are very beneficial in such tumors (Herceptin). They carry a poor prognosis.
- *Basal-like/Triple Negative type*: They comprise 10–20% of all breast cancers. Occur in younger age groups and in African-American communities. They are commonly seen in people with BRCA 1 ad p53 mutations. They have a very high rate of recurrence and respond to chemotherapy.

**Table:** 

Molecular subtypes	ER	PR	Her2neu	Ki-67 (%)
Luminal A	+	+	−	< 14
Luminal B	+	+	+/−	≥ 14
Her2neu positive type	−	−	+	≥ 14
Basal like/triple negative	−	−	−	≥ 14

This study aims to look into beta hCG as another potential indicator of the same.

The secretion of Beta hCG is most predominantly associated with pregnancy, thus it is important to study the relationship between pregnancy and breast carcinoma. It is a well-established fact that higher and earlier parity imparts a protective factor in breast cancer. There are four main theories explaining how pregnancy may provide protection against breast cancer:

1. *Hormonal Fluctuations*: Pregnancy involves fluctuations in hormone levels, such as estradiol, prolactin, and growth hormone. These hormonal changes may be associated with breast cancer risk and could potentially provide some protective effect.
2. *Differentiation of Epithelial Cells*: The extensive development of terminal ductal lobuloalveolar units during pregnancy leads to differentiation of epithelial cells. This differentiation may persist even after involution, resulting in a subset of cells that are less prone to malignant transformation.
3. *Influence on Breast Stem Cells*: Parity (having given birth) appears to have an impact on breast stem cells, although the exact mechanisms are not yet fully understood.
4. *Estrogen Exposure and ER*+ *Tumors*: Pregnancy's protective effect is particularly evident in estrogen receptor-positive (ER+) tumors. The duration of estrogen exposure has been correlated with breast cancer risk, suggesting that the effects of estrogen on the breast, possibly through interactions with hormone-sensing and stem cells or changes in the response of hormone-sensing cells to estrogen, contribute to the protective effect.

Our study on Beta hCG is specifically related to the fourth theory, which highlights the involvement of estrogen exposure and its interaction with hormone-sensing cells^7,8^. The expression of luteinizing hormone (LH)/human chorionic gonadotrophin (hCG) receptor in mammary tissue plays a role in the action of placental hCG. This receptor is primarily located in ovarian corpus luteum cells, where it binds LH and stimulates progesterone production^9,10^. LH/hCG receptors have been found in both normal mammary epithelium and breast cancer cases^11,12^. Interestingly, the expression of these receptors is higher in normal breast tissue compared to malignant breast tissue, indicating a potentially more pronounced effect of hCG in normal breast tissue. Beta hCG and LH may contribute to pro-mitogenic effects that can lead to the malignant transformation of normal cells^13–15^.

A 2008 study published in the Journal of Cancer Research and Clinical Oncology explored the potential role of Beta-hCG/LH-R in breast cancer development. The study used immunofluorescence to examine the expression of the beta hCG/LH-R system in breast cancer samples^16^. Among the 70 samples analyzed, it was observed that 21.6% of the pre-invasive samples and 70.4% of the adjacent invasive samples exhibited beta hCG/LH-R expression, indicating a significant increase in invasive tumors compared to pre-invasive ones. In vitro studies have demonstrated that the Beta-hCG/LH/LH-R system can both stimulate and inhibit the growth of ductal epithelium. Furthermore, recent evidence suggests that a common Beta-hCG/LH-R gene variant may affect disease-free survival in breast carcinoma patients, supporting the hypothesis of an upregulation of Beta-hCG/LH-R expression in breast cancer cases. This suggests a potential role of beta-hCG/LH-R in promoting breast carcinogenesis under specific circumstances^16^.

Research shows that hCG could potentially be a hormone exerting anti-tumorigenic effect on breast cancer. However, this hormone appears to have a paradoxical role in cases of breast carcinoma. It has been concluded that whereas placental hCG appears to have a protective effect on the breast by preventing transformation to malignancy, ectopically produced beta hCG is associated with poor prognosis when seen in carcinomas. Thus, the two appears to have opposite effects on breast tumours^1^.

Certain studies have hypothesized this functionality of beta hCG in the tumour progression. Beta hCG seems to show bimodal activity in breast cancers. Multiparity is known to offer protection against breast carcinoma and this has largely been attributed to be due to the beneficial effects of beta hCG. Whereas there have been studies indicating the ‘pro carcinogenic’ properties of beta hCG as well^1,17^.

A study by Janssens et al., substantiating this bimodal activity of beta hCG in breast carcinoma was conducted in 1998 and 1999 in two phases, where in phase I the inhibitory effects of recombinant hCG (rhCG) was tested on primary breast cancer. 25 women belonging to the postmenopausal age group, with newly diagnosed breast cancers measuring greater than 1.5 cm underwent a biopsy of the lesion, before being subjected to randomization to receive either 500 g rhCG (n = 20) or placebo. These women then underwent surgery after 2 weeks and the tissues obtained were evaluated for IHC markers, morphology and biochemical changes. In phase II, the clinical efficacy of rhCG was studied through an open-label single centre study in 13 women of postmenopausal age group having metastatic breast carcinoma. It was observed that in primary breast carcinoma without metastasis, administration of rhCG resulted in a significant decrease in the proliferative index (Ki67). However, such an effect was not seen in the patients who were administered placebo. rhCG also reduced the levels of ER and PR but did not appear to have any effect on the hormonal level of estradiol, progesterone, inhibin or FSH. However, in phase II (women with metastatic breast cancer), administration of rhCG led to four patients (31%) having progressive disease after two months (sixty days), seven patients (54%) had stable disease and two patients had a decrease of the soft tissue localizations for more than 50% of the initial diameters^17^.

The above study gives us insight on the use of Beta hCG as a therapeutic modality and its use in the prevention of breast cancer. From the results of the above the study its use has been successful in postmenopausal metastatic breast cancer. Thus, it has been demonstrated that beta hCG does have activity on breast cancer cells and it can be used as a treatment modality in patients with breast cancer. Furthermore, its use in the prevention of development of breast cancer by targeting the LH/hCG receptor is still under trial. Targeting such a receptor can thus have a dramatic impact in the prevention of the disease^17^.

The presence of beta hCG in breast tumors has been associated with both poor prognosis and potential preventive effects against breast cancer^18^. Some studies suggest that exogenous administration of beta hCG, mimicking a state of pregnancy, could be used for breast cancer prevention. Additionally, targeting beta-hCG-expressing tumor cells may offer a therapeutic approach for breast cancer treatment. Approximately 13% of breast cancer cases have been found to express beta hCG. The detection of beta hCG in breast carcinomas can serve as a marker for malignancy. Based on the protective role proposed for beta hCG against breast cancer, there are advocates for the prophylactic administration of beta hCG to non-pregnant women as a preventive measure. Studies conducted on mice have shown that a beta hCG-based tumor vaccine provided protection against breast tumors^18^.

In a study conducted by Sengodan et al., the expression of beta hCG in breast carcinoma was examined. The study revealed that beta hCG expression is related to the BRCA1 status, with overexpression observed in cases of breast carcinoma with BRCA1 mutation^19^. It was further observed that beta hCG promotes migration and invasion in breast cancer cells, particularly those with BRCA1 mutation. The results led to the deduction that BRCA1 suppresses the expression of beta hCG, and its mutation results in increased beta hCG expression. Notably, patients with BRCA1 mutation showed elevated beta hCG levels despite negative ER, PR, and HER2/neu status. Based on these findings, the study proposed the potential use of a vaccine containing beta hCG antibodies as a treatment approach for patients with BRCA1-mutated breast cancers. Consequently, ongoing trials are exploring the development of immunization strategies utilizing beta hCG antibodies^19^.

According to the Hellman hypothesis, breast cancer is a systemic disease that spreads early, with cancer cells present in the peripheral blood. However, the presence of these cells does not guarantee the development of metastasis, as only a small percentage will successfully seed into distant organs^20,21^. This understanding supports the rationale for measuring serum beta hCG levels in women diagnosed with breast carcinoma. Cancerous breast tissue expresses beta hCG receptors, and detecting cancer cells expressing beta hCG in the peripheral blood can aid in early detection of breast cancer. However, the challenge lies in detecting these circulating cells, as they may be present in low numbers. To overcome this, the reverse transcriptase-polymerase chain reaction (RT-PCR) technique can be used to amplify the mRNA expression in breast cancer cells, increasing the sensitivity of detection in the early stages of the disease.

To substantiate aforementioned theory, a study was published in the Journal of the Polish Biochemical Society and of the Committee of Biochemistry and Biophysics Polish Academy of Sciences in 2004 where RT-PCR was used to amplify the breast cancer cells in peripheral blood, thereby aiding in its detection^20^. In contrast, in our study, we analyze the levels of ER, PR, HER2neu and ki-67 and correlate it with beta hCG levels.

Thus far we have discussed evidence which is based on the immunohistochemical demonstration of beta hCG in breast cancer cells. This study however, strives to demonstrate serum beta hCG detection.

In a 2019 cross-sectional study published in the Journal of Experimental Therapeutics and Oncology, the effects of beta hCG and its receptor status were examined in relation to clinic-pathological characteristics in women with breast carcinoma^21^. The study involved 57 patients, and it was found that patients with tumor sizes ranging from 2 to 5 cm and those with a higher clinical stage at presentation exhibited higher expression of the beta hCG receptor. The study also observed a direct correlation between beta hCG levels and other tumor characteristics such as histopathology, tumor size, nodal status, and grade. Specifically, higher tumor size and advanced disease staging were associated with higher levels of beta hCG^22^.

This has a direct impact on the utility of this marker as the presence and early recognition of beta hCG can be used to prognosticate the disease by clubbing it along with the other commonly used tumour markers of ER, PR, Her2neu and Ki67 thereby increasing the screening of such patients and aiding in the earlier detection of breast cancer.

The current screening modalities of breast carcinoma involve testing of such markers on the breast tissue, thereby subjecting a woman to an invasive procedure of tru cut biopsy. The measurement of serum beta hCG levels can be incorporated with other routinely done serum investigations and if detected early could significantly improve the prognosis of such woman by giving them an early warning sign. This study and its demonstration of serum beta hCG is suggestive of the breast cancer cellular expression of hCG being translated into the patient’s blood, and is thus is an important foundation for our study.

Majority of the evidence discussed above, however, seems to point firmly at the expression of beta hCG on breast cells during malignancy of the breast. This study therefore focuses on attempting to find the relative occurrence of this translating into the increase of serum beta hCG levels and to draw a correlation between this expression and the specific tumour stage and evaluating the possibility of beta hCG as a tumour.

In our study, 200 women belonging to 18–65 years of age with malignant palpable breast lesions were evaluated for serum Beta hCG levels in an attempt to find a correlation between Beta hCG and

- Various stages of breast carcinoma in terms of TNM staging.
- Age of the patient.
- Different histological types of breast carcinoma.

All the women evaluated were also subjected to a histopathological diagnosis with a full metastatic workup and a TNM staging. They were also assessed for the regular tumour markers—ER, PR. HER2neu, and ki67.

There was no increase found in the serum Beta hCG, in women with breast malignancies, in our study, however, a pattern was observed amongst the negative results.

In our study, serum Beta hCG levels were not found to have an association with the histological type of breast carcinoma, leading to the inference that changes in Beta hCG values were independent of the histological subtype. Similarly, there was no correlation observed between Beta hCG levels and age of the patient with breast carcinoma.

Amongst the 200 women evaluated, the p value for Beta hCG was found to have significance association with the stage of the disease, with respect to TNM classification of breast carcinoma, with a p value of 0.036. The serum Beta hCG level showed a rising trend with progression in the stage of the disease; that is, as the stage worsened.

We also found that, of the 20 women who had M1 disease, each one had a serum Beta hCG value of > 2mIU/mL, thereby indicating that metastatic disease had a higher value of serum Beta hCG as opposed to non-metastatic disease. There was also a direct correlation noted between the T stage of the disease and Beta hCG, with increased levels of serum Beta hCG being observed in T3 and T4 stages and with a p value of 0.006 which is highly significant. It was also found to correlate with the N stage, with a rise seen in serum Beta hCG value with a progressing N stage of the disease. Similar findings were also observed in Stage 3 and Stage 4 of breast carcinoma, with increased levels of serum Beta hCG.

We also tried to find a correlation between Beta hCG and the commonly used tumour markers such as ER, PR, HER2neu and ki67 in order to use as an adjunct for prognostication of the disease. Even though Beta hCG was not found to be correlating to the ER, PR, HER2neu, there was a strong association noted with ki67.

Ki67 can be classified as low or high risk based upon its expression on tumour cells. A < 15% value of ki67 is considered as low risk^23,24^. When correlating ER, PR, HER2neu with ki67, 116 women were found to be high risk for ki67, 90 out of which were found to have a serum Beta hCG value of > 2mIU/mL, thereby indicating that 75% of such women had a strong correlation between ki67 tumour marker and Beta hCG, giving a p value of 0.33 which is significant.

## Conclusions

Evaluation of Beta hCG levels in blood is a faster, cost effective and easy to execute method as compared to the detection of other tumour markers and hence can be used to assess the prognosis in patients. However, the inability to evaluate Beta hCG in breast tissue due to obtaining breast tissue sample being an invasive procedure and the associated expenses has been a limitation to our study. Moreover, the presence of other high Beta hCG states such as pregnancy, tumours could affect the study outcome.

Although the serum beta hCG value was negative in all women with breast carcinoma, i.e. < 5mIU/mL, a rising trend was observed in its levels in relation to the progression of stage of disease, with higher values of serum Beta hCG seen in the later stages of the disease. This trend was also observed in association with ki67 tumour marker, with serum Beta hCG levels being > 2mIU/mL in women found to have ki67 value of ≥ 15% hence leading to the conclusion that the higher levels of serum Beta hCG, were associated with a graver prognosis.

Even though Beta hCG cannot be used as tumour marker, its high significance with respect to TNM staging and ki67 tumour marker can be used to prognosticate the severity of breast carcinoma in women with palpable breast malignancies and to predict the outcome of the disease in association with other tumour markers such as ER, PR and HER2neu.

### Future course of action

1. This study can be broadened to include tissue assay of beta hCG and its correlation with the histopathological subtypes of breast carcinoma.
2. To assess the use of beta hCG in non-malignant breast lump.
3. For the development of hCG bases tumour vaccines and hCG targeted therapy for prevention and treatment of breast carcinoma.

## Data Availability

The datasets used and/or analyzed during the current study are available from the corresponding author on request.
